# Supplementary material for: Causal Relationship between Aging and Anorexia Nervosa: A White-Matter-Microstructure-Mediated Mendelian Randomization Analysis
Source: Biomedicines. 2024 Aug 16;12(8):1874. doi: 10.3390/biomedicines12081874 (PMC11351342; doi:10.3390/biomedicines12081874)
Supplement: Supplementary file 1 [file biomedicines-12-01874-s001.zip › Supplementary Figure/Figure S4/Singe SNP plot of MR analysis for TL to ubm-b-2091.pdf]

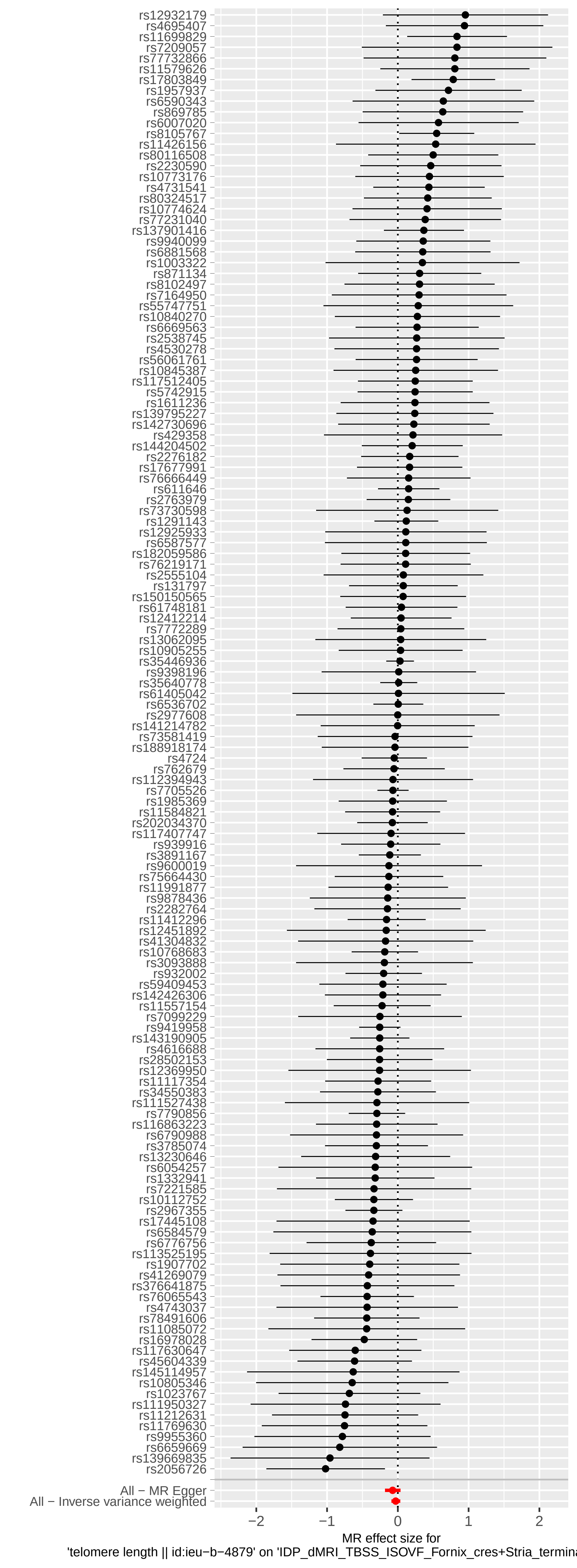
rs12932179
rs4695407
rs11699829
rs7209057
rs77732866
rs11579626
rs17803849
rs1957937
rs6590343
rs869785
rs6007020
rs8105767
rs11426156
rs80116508
rs2230590
rs10773176
rs4731541
rs80324517
rs10774624
rs77231040
rs137901416
rs9940099
rs6881568
rs1003322
rs871134
rs8102497
rs7164950
rs55747751
rs10840270
rs6669563
rs2538745
rs4530278
rs56061761
rs10845387
rs117512405
rs5742915
rs1611236
rs139795227
rs142730696
rs429358
rs144204502
rs2276182
rs17677991
rs76666449
rs611646
rs2763979
rs73730598
rs1291143
rs12925933
rs6587577
rs182059586
rs76219171
rs2555104
rs131797
rs150150565
rs61748181
rs12412214
rs7772289
rs13062095
rs10905255
rs35446936
rs9398196
rs35640778
rs61405042
rs6536702
rs2977608
rs141214782
rs73581419
rs188918174
rs4724
rs762679
rs112394943
rs7705526
rs1985369
rs11584821
rs202034370
rs117407747
rs939916
rs3891167
rs9600019
rs75664430
rs11991877
rs9878436
rs2282764
rs11412296
rs12451892
rs41304832
rs10768683
rs3093888
rs932002
rs59409453
rs142426306
rs11557154
rs7099229
rs9419958
rs143190905
rs4616688
rs28502153
rs12369950
rs11117354
rs34550383
rs111527438
rs7790856
rs116863223
rs6790988
rs3785074
rs13230646
rs6054257
rs1332941
rs7221585
rs10112752
rs2967355
rs17445108
rs6584579
rs6776756
rs113525195
rs1907702
rs41269079
rs376641875
rs76065543
rs4743037
rs78491606
rs11085072
rs16978028
rs117630647
rs45604339
rs145114957
rs10805346
rs1023767
rs111950327
rs11212631
rs11769630
rs9955360
rs6659669
rs139669835
rs2056726
All − MR Egger
All − Inverse variance weighted
−2
−1
0
1
2
MR effect size for
'telomere length || id:ieu−b−4879' on 'IDP_dMRI_TBSS_ISOVF_Fornix_cres+Stria_termina
